# Sex Differences in the Presence and Severity of Alcohol Hangover Symptoms

**DOI:** 10.3390/jcm8060867

**Published:** 2019-06-17

**Authors:** Albertine E. van Lawick van Pabst, Lydia E. Devenney, Joris C. Verster

**Affiliations:** 1 Division of Pharmacology, Utrecht University, 3584 CG Utrecht, The Netherlands; albertinevanlawick@live.nl; 2 School of Psychology, Life and Health Sciences Ulster University, Londonderry BT52 1SA, Northern Ireland, UK; Devenney-l2@ulster.ac.uk; 3 Centre for Human Psychopharmacology, Swinburne University, Melbourne VIC 3122, Australia

**Keywords:** alcohol, hangover, symptoms, sex differences, presence, severity

## Abstract

Studies have demonstrated significant sex differences in alcohol intoxication effects. In contrast, the majority of studies on the alcohol hangover phase did not investigate sex differences. Therefore, the current study examined possible sex differences in the presence and severity of alcohol hangover symptoms. Data from *n* = 2446 Dutch students (male = 50.7%, female = 49.3%) were analyzed. They reported the presence and severity of 22 hangover symptoms experienced after their past month heaviest drinking occasion. Subjects were categorized according to their estimated peak blood alcohol concentration (eBAC) and presence and severity of the hangover symptoms were compared between men and women. In the lowest eBAC group (0% ≤ eBAC < 0.08%), no significant sex differences were found. In the subsequent eBAC group (0.08% ≤ eBAC < 0.11%), severity of nausea was significantly higher in women than in men. In the third eBAC group (0.11% ≤ eBAC < 0.2%), women reported higher severity scores on nausea, tiredness, weakness, and dizziness than men. Men reported the presence of confusion significantly more often than women, and women reported the presence of shivering significantly more often than men. In the fourth eBAC group (0.2% ≤ eBAC < 0.3%), women reported higher severity scores on nausea and tiredness than men. In the highest eBAC group (0.3% ≤ eBAC < 0.4%), no significant sex differences were found. In conclusion, across the eBAC groups, severity scores of nausea and tiredness were higher in women than in men. However, albeit statistically significant, the observed sex differences in presence and severity of hangover symptoms were of small magnitude, and therefore, have little clinical relevance.

## 1. Introduction

The alcohol hangover is defined as the combination of mental and physical symptoms that are experienced the day after an episode of heavy alcohol consumption, starting when blood alcohol concentration (BAC) approaches zero [1]. Alcohol hangovers may negatively impact people’s psychological and physical well-being by increasing accidents and injury [2,3] and impairing daily activities such as driving a car [4,5] or riding a bicycle [6].

Sex differences in acute alcohol effects have been well documented. Females have more body fat and less water than men of the same body weight [7]. Since alcohol is dispensed in body water, women reach higher BAC levels than men despite consuming an identical number of alcohol units [7]. Moreover, women usually have increased bioavailability and faster disappearance rates than men [8]. Also, alcohol appears to impair cognitive and psychomotor functioning in women more than in men [9,10].

Research usually shows that men consume more alcohol than women on a single drinking session (e.g., [11]). Therefore, in most observational and naturalistic studies men have higher BACs and experience more negative alcohol related consequences and performance impairment then women. However, studies employing the self-rating of the effects of alcohol (SRE) form show that at the same BAC levels, women usually are more sensitive to alcohol effects than men [12]. Lower scores on the SRE imply that fewer alcoholic drinks are needed to achieve a certain effect, meaning that subjects are more sensitive to alcohol. Usually, men have significantly higher SRE scores than women [13], suggesting that at the same BAC level men are less sensitive to (the adverse effects of) alcohol compared to women. As sex differences in pharmacokinetics and pharmacodynamics sex have been observed after acute alcohol intake, it is important to further investigate possible sex differences in the presence and severity of alcohol hangover effects.

A search of the literature revealed that the majority of studies on alcohol hangovers did not investigate possible sex differences. In fact, many older studies included men only. For example, until 2004, studies on the pathology and physiological correlates of alcohol hangover were solely conducted in male subjects [14,15,16,17,18,19,20,21,22,23,24,25,26,27,28].

Also, many studies investigating cognitive impairments that may accompany the alcohol hangover used samples that consisted of men only [29,30,31,32,33,34,35,36,37,38,39,40,41,42,43,44]. In addition, some studies have focused on women only [45], while others did not mention the sex of their participants [46,47]. Moreover, several studies on cognitive performance included both men and women but omitted any statistical analysis on possible sex effects [6,48,49,50,51,52,53,54,55], leaving it unknown whether sex differences were either absent or not investigated. Up to now, six controlled studies examining performance on a variety of cognitive, psychomotor, and memory tests found no significant sex differences on any of the administered tests [56,57,58,59,60,61], and two other studies reported no significant sex differences in driving performance [4,62]. In experimental studies where sex-adjusted dosing was used, with few exceptions [58], usually no sex differences in the presence and severity of hangover symptoms was found [60,63]. Most commonly, however, possible sex differences were not investigated. Also, with few exceptions [64,65], survey studies usually did not report on sex differences.

Insight into possible sex differences in the symptomatology of the alcohol hangover may help to increase our understanding of the pathology of the alcohol hangover and be of guidance in the development of sex-tailored alcohol hangover treatments, targeting those core symptoms that are characteristic for the hangover state in relation to different peak eBAC levels. The aim of the current study was, therefore, to systematically investigate possible sex differences in the presence and severity of alcohol hangover symptoms at various estimated blood alcohol concentration (eBAC) ranges. The latter is a novel approach compared to previous research but differentiating BAC levels is important, as sex-effects may be differential at high or low BACs. Given the pharmacokinetic and pharmacodynamic sex differences in acute alcohol effects, sex differences in the presence and severity of hangover symptoms were also expected to be present during the hangover state.

## 2. Methods

Data from two previous surveys on alcohol hangovers were combined [66,67]. The combined dataset consisted of *n* = 2446 Dutch students (male = 50.7%, female = 49.35%), with an age range of 18 to 30 years old. For their past month’s heaviest drinking occasion, they reported the number of alcoholic drinks they consumed and the start and stop time of drinking. The estimated peak blood alcohol concentration (eBAC) for this drinking occasion was calculated by applying an adapted Widmark equation [68]. As the eBAC formula includes data on weight, sex, duration of drinking, and the amount of alcohol consumed, eBAC can be regarded as the most overall alcohol consumption parameter. The severity of 22 individual hangover symptoms was scored on an 11 point scale ranging from 0 (absent) to 10 (extreme) [69]. The 22 individual hangover symptoms are a combination of those listed in the three most commonly used hangover symptom scales [70,71,72], and include headache, nausea, concentration problems, regret, sleepiness, heart pounding, heart racing, vomiting, tiredness, shivering, clumsiness, weakness, dizziness, apathy, sweating, stomach pain, confusion, sensitivity to light, thirst, anxiety, depression, and reduced appetite.

### Statistical Analysis

Data were analyzed using SPSS version 25. Subjects who reported no hangover and those with an eBAC of 0.40% and higher were omitted from the analysis. The presence of each of the 22 hangover symptoms was calculated (i.e., the % of subjects with a score >0). Mean (SD) hangover symptom severity was calculated only for those subjects reporting a particular hangover symptom (i.e., excluding scores of 0). The presence and severity of individual hangover symptoms were compared between men and women using the nonparametric Chi-squared test (presence, comparing percentages) and the Mann–Whitney U test (severity, comparing means), respectively. A Bonferroni correction was applied to adjust for multiple comparisons. Sex differences were considered statistically significant if *p* < 0.002.

Since alcohol consumption levels may significantly differ between men and women, as well as the corresponding presence and severity of hangover symptoms, subjects were grouped according to their estimated blood alcohol concentration (eBAC): (a) 0% ≤ eBAC < 0.08%, (b) 0.08% ≤ eBAC < 0.11%, (c) 0.11% ≤ eBAC < 0.2%, (d) 0.2% ≤ eBAC < 0.3% and (e) 0.3% ≤ eBAC < 0.4%. The lower cut-off values (a) correspond to common legal limits for driving, whereas ≥0.11% (d) corresponds to the Alcohol Hangover Research Group proposed eBAC limit needed to provoke a hangover per se [73]. For the current study, participants with an eBAC of ≥0.4 were considered outliers (>2 SD from the mean) and excluded from the data analysis.

## 3. Results

From the *n* = 2446 subjects that completed the survey, those who reported no past month hangover were omitted from the analysis (*n* = 681, 27.6%). Thus, data on the presence and severity of hangover symptoms from *n* = 1765 subjects (*n* = 895 men and *n* = 870 women) were compared. Demographics of the sample are summarized in Table 1.

Women reported consuming significantly less alcohol per week than men. Also, on the latest past month drinking occasion that resulted in a hangover, women reported consuming significantly fewer alcoholic drinks then men. Overall hangover severity and eBAC on the latest past month drinking occasion that resulted in a hangover did not significantly differ between men and women. 

The overall results (0 ≤ eBAC < 0.4%, see Table 2) show that women reported significantly higher hangover symptom scores than men for nausea, sleepiness, being tired, vomiting, weakness, sensitivity to light, and dizziness. Further, the presence of shivering was reported significantly more often by women, whereas the presence of heat racing, confusion, and sweating were reported significantly more frequently by men. Subjects were further categorized according to their eBAC. The results for the sex comparisons for each eBAC range are summarized in Table 3, Table 4, Table 5, Table 6 and Table 7.

In the lowest eBAC group (0% ≤ eBAC < 0.08%, see Table 3) no significant differences were found between men (*n* = 91) and women (*n* = 68). In the subsequent eBAC group (0.08% ≤ eBAC < 0.11%, see Table 4), nausea was significantly more severe in women (*n* = 95) compared to men (*n* = 93). No other significant sex differences were found. Below an eBAC of 0.11%, the presence and severity of hangover symptoms were relatively low. This was expected given the relative low alcohol intake, and in line with the previous consensus paper of the Alcohol Hangover Research Group which stated that an eBAC < 0.11% is insufficient to produce a hangover per se. A minority of the total sample had an eBAC below 0.11%, which had an approximate equal sex distribution (18.7% of all women and 20.6% of all men).

**Figure 1:**
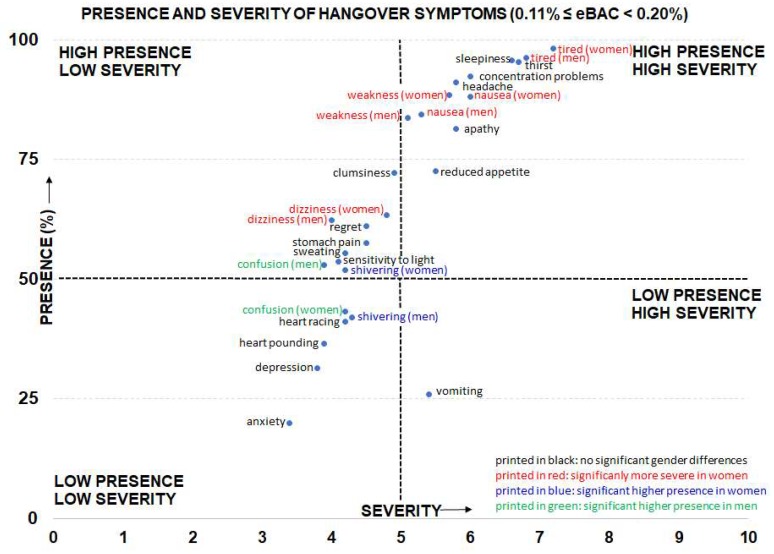
Presence and severity of individual hangover symptoms after alcohol consumption and reaching an eBAC of 0.11% ≤ eBAC < 0.2%. Sex differences are significant if *p* < 0.002. Abbreviation: eBAC = estimated blood alcohol concentration.

Table 5 summarizes the results of the largest group of drinkers (*n* = 403 men and *n* = 383 women). Their eBAC ranged from 0.11% ≤ eBAC < 0.2%. In women, severity scores on nausea, tiredness, weakness, and dizziness were significantly higher than in men. In addition, confusion was significantly more often reported by men and shivering was significantly more often reported by women. All other sex comparisons did not reach statistical significance. Figure 1 gives an overview of the presence and severity of individual hangover symptoms for the eBAC range 0.11% ≤ eBAC < 0.2%. It is evident from Figure 1, that the observed significant sex differences are of modest magnitude. Except for dizziness, sex differences in symptom severity were seen only among symptoms that had high presence ratings. Figure 1 also reveals that some symptoms had a low severity and a low presence (e.g., anxiety and depression), whereas other symptoms had a high severity and high presence (e.g., sleepiness, headache, concentration problems, apathy). In this regard, vomiting was an interesting hangover symptom: vomiting had a low presence, but when it occurred its severity was relatively high.

Table 6 summarizes data from subjects within the eBAC range 0.2% ≤ eBAC < 0.3% (*n* = 244 men and *n* = 264 women). In women, severity scores on nausea and tiredness were significantly higher than among men. All other sex comparisons did not reach statistical significance.

Finally, Table 7 summarizes data from subjects within the eBAC range 0.3% ≤ eBAC < 0.4% (*n* = 62 men and *n* = 62 women). For this eBAC range, no significant sex differences were observed.

## 4. Discussion

**Figure 2:**
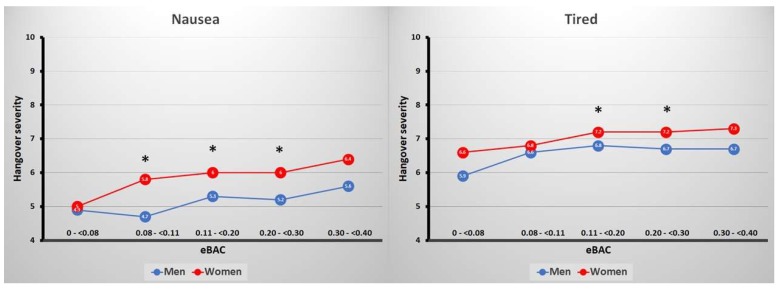
Severity scores for nausea and being tired for men and women across eBAC ranges. Significant differences (*p* < 0.002) are indicated by *. Abbreviation: eBAC = estimated blood alcohol concentration.

The current analyses showed that some sex differences in the presence and severity of hangover symptoms were observed. These were especially evident at the eBAC levels ranging from 0.11% ≤ eBAC < 0.2%. In the lowest eBAC group (0% ≤ eBAC < 0.08%), no significant sex differences were found. In the subsequent eBAC group (0.08% ≤ eBAC < 0.11%), severity of nausea was significantly higher in women than in men. In the third eBAC group (0.11% ≤ eBAC < 0.2%), women reported higher severity scores on nausea, tiredness, weakness, and dizziness than men. Men reported the presence of confusion significantly more often than women, and women reported the presence of shivering significantly more often than men. In the fourth eBAC group (0.2% ≤ eBAC < 0.3%), women reported higher severity scores on nausea and tiredness than men. The fifth eBAC group (0.3% ≤ eBAC < 0.4%), no significant sex differences were found. However, the observed sex differences were of modest magnitude and have little clinical relevance. This is illustrated in Figure 2 for the most frequently reported sex differences in hangover symptom severity, i.e., nausea and being tired. The magnitude of the differences in symptom severity scores for nausea and being tired, albeit statistically significant, across eBAC ranges were always below 1 on a scale ranging from 0 (absent) to 10 (extreme). Figure 2 further shows that symptom severity scores did somewhat increase when eBAC became higher. For example, the difference between the lowest eBAC group (<0.08%) and the highest eBAC group (0.3%–<0.4%) for nausea were +0.7 in men and +1.4 in women, and for being tired +0.8 in men and +0.7 in women. Thus, the present analyses suggest that men and women experience hangovers in comparable ways.

### 4.1. Alcohol Sensitivity during Intoxication and during the Hangover State

The absence of relevant sex differences in the presence and severity of hangover symptoms contrasts with the various sex differences that are commonly observed in pharmacokinetics and pharmacodynamics during the alcohol intoxication phase. Thus, there seems to be a discrepancy between sex differences observed during alcohol intoxication (in previous research) and the absence of relevant sex effects during the hangover state (found in the current study). Previous research has addressed this issue. For example, Piasecki and colleagues [74] reported that subjects with lower alcohol sensitivity appear to be more resistant to experiencing hangovers at a given number of drinks. However, as these drinkers often consume more alcohol than those with high alcohol sensitivity, they on average experience more hangovers during an arbitrary monitoring period. Unfortunately, the association with alcohol sensitivity and hangover severity was not assessed in this study.

Rohsenow et al. [75] did not administer the SRE but instead used an alcohol intoxication rating as a proxy measure for the sensitivity to acute alcohol effects. They found that hangover severity, as assessed with the AHS (Acute Hangover Scale), was positivity correlated with this intoxication rating. In this study, about one-third of the studied sample reported an absence of hangover after consuming alcohol to reach a peak BAC of 0.12%. As hangover insensitivity was associated with reports of being less intoxicated, the authors suggested that hangover sensitivity and intoxication sensitivity may be related to each other. In an earlier study, Ylikhari et al. [15] also found that, despite consuming the same amount of alcohol (1.5 g/kg), those who reported higher subjective intoxication levels also experienced more severe hangovers.

In a naturalistic study comprising of an alcohol and alcohol-free test day, Hogewoning et al. [69] administered the SRE and compared drinking behavior and hangover effects in both hangover-sensitive drinkers and hangover-resistant drinkers. Although both groups consumed a comparable amount of alcohol, achieving a peak BAC around 0.17%, the hangover resistant group reported not experiencing a hangover while the sensitive group did. Hogewoning et al. [69] found that the early life SRE score and total SRE score were significantly lower in women compared to men. However, in contrast to Piasecki et al. [74], SRE scores did not significantly differ between hangover-sensitive and hangover-resistant drinkers, and the SRE scores did not significantly correlate with hangover severity. Taken together, these findings suggest that sex differences in alcohol sensitivity seen during the intoxication phase may be unrelated to the sensitivity for alcohol effects expected during the hangover state. There may be several explanations for this observation.

Firstly, there is a time lag between the intoxication phase and the hangover phase. After stopping drinking, usually a period of sleep follows of which the quality and duration has shown to be associated with hangover severity [76,77]. Secondly, the acute alcohol effects are mainly caused by a direct presence of alcohol in the blood. Subsequently, alcohol can exert its effects virtually everywhere in the body, including the brain as it easily crosses the blood–brain barrier. In contrast, the hangover state starts when blood alcohol concentrations approach zero, and in many cases BAC readings are zero. Finally, although there is some overlap between the presence and severity of symptoms that are experienced during the intoxication and hangover phase, there are also several clear differences. Van Schrojenstein Lantman et al. [1] reported that the most common reported combination of symptoms describing the alcohol hangover comprised of the following: nausea, headache, tiredness, and apathy. These symptoms showed the highest associations between each other. Except for nausea, these are symptoms that are usually not reported to be present or have high severity ratings during the intoxication phase of drinking. Taken together during the intoxication phase, sex differences in the presence and severity of intoxication effects are common. However, in contrast, sex differences in symptomatology during the alcohol hangover are of limited relevance.

### 4.2. Implications

The current findings have several implications. Firstly, our findings do not suggest that results from studies conducted in men can be directly extrapolated to women, or that an equal sex balance would not be necessary. Although the presence and severity of hangover symptoms do not seem to vary much between men and women, their impact on mood and performance may be considerably different. Therefore, we advocate an equal sex balance in future hangover studies. Secondly, the current findings may have implications for drug development. In past research, the efficacy and safety of potential hangover treatments were often not investigated in women (e.g., [33,78,79,80,81,82,83]). Currently, most marketed hangover products lack sound scientific support that confirms their efficacy and safety [84,85], and no effective treatment has been developed yet. The current analyses suggest that in order to develop an effective and safe hangover treatment, similar symptoms should be targeted for both sexes, as they usually not differ in presence and severity. Of note, in both sexes, with increasing BAC levels the presence and severity of hangover symptoms increases to some extent. Therefore, dose-ranging studies to demonstrate efficacy and safety at various BAC levels (e.g., low, moderate, high intake) may be worthwhile to investigate. Given the potential negative impact of hangover symptoms on performance of daily activities such as driving and job performance [2,3,4,5,6], developing an effective hangover treatment could be useful. Previous research has shown that the vast majority of drinkers indicate that experiencing hangovers does not affect future drinking behavior [86], and that if an effective hangover treatment would be available this would not significantly alter their drinking behavior [87]. However, recent research did associate experiencing hangovers with having significantly more future drinking days and alcohol problems [88]. Therefore, more research should address the relationship between experiencing hangovers or their treatment with increased risk of developing alcohol use disorder.

As for all clinical trials investigating psychopharmacological treatments [89,90,91], it is also critical to examine the efficacy and safety of new hangover treatments in both men and women. Although the targeted hangover symptoms may have the same presence and severity, the safety, adverse effects, and efficacy of the hangover treatment itself may show significant sex differences. Even in the absence of a clear a priori explanation for expecting sex differences it is vital to examine possible dissociations in pharmacokinetics and pharmacodynamics of a hangover treatment.

From Figure 1, it is clear that some symptoms are high in both presence and severity. These can be regarded as core symptoms of the hangover state, and it is suggested that further research focuses on these symptoms. In this context, there are currently three hangover symptom scales in use that all comprise ratings of different hangover symptoms to sum up to an overall hangover score [70,71,72]. A new analysis, taking into account both the presence and severity of symptoms, and perhaps their impact on cognitive and physical functioning and mood [67], can be useful for the development of a new hangover scale. Finally, some symptoms such as anxiety and depression have both low presence and severity scores. This is a consistent finding in scientific literature, and although these symptoms are included in the Hangover Symptoms Scale (HSS) [71], it can be questioned if they are true hangover symptoms or should be considered as individual subject characteristics that are unrelated to alcohol consumption and its aftereffects.

### 4.3. Strengths and Limitations

Strengths of the current analysis include its large sample size, and the fact that subjects were stratified according to their estimated peak BAC level. The latter was critical to obtain a fair comparison between effects observed in men and women.

A limitation of the survey data consisted of retrospective ratings of hangover symptoms, experienced during their latest past month alcohol hangover. Therefore, in theory, impairments in accurate recall may have biased these results. However, there is also no reason to assume why possible recall bias would be different in men and women, or more pronounced for one hangover symptom over the others.

Second, the participants in the surveys consisted of students, aged 18 to 30 years old. This may limit the generalizability to other age groups or non-student samples. Hangover data on subjects aged 40 and beyond are virtually absent in the scientific literature. One study did report a shift in the presence and severity of hangover symptoms with progressing age [92]. This population-based Danish Health Examination Study (DANHES) gathered data from over 50,000 adults and showed that with increasing age, the presence of hangovers significantly decreased, and in a comparable rate for men and women. Both men and women reported fewer hangover symptoms (thirst, exhaustion, headache, dizziness, no appetite, stomach pain, nausea, heart racing, and vomiting) with increasing age. Although several reasons for this observation can be hypothesized (e.g., tolerance, changes in sensitivity, and alcohol metabolism), the most likely reason for the age-progressing decline in hangover frequency is the accompanying age-related reduction in the amount of (alcohol consumed on) heavy drinking sessions [93]. Although no formal testing was presented by Tolstrup et al. [92], inspection of the odds ratios for “(almost) always experiencing a hangover symptom” suggests that there are no clinically relevant sex differences across age groups (Tolstrup et al. [92], Table 3, page 469). Future research should further examine possible sex differences in hangover symptomatology in other age groups.

Third, we investigated the 22 hangover symptoms that are included in the three commonly used hangover scales [70,71,72]. Research has shown that there are several other hangover symptoms that we could have considered in the current study. For example, Penning et al. [66] identified 47 hangover symptoms in the literature. However, most of the symptoms that were omitted from the three hangover scales (e.g., nystagmus or tinnitus) are very infrequently reported and their severity scores are usually low. Hence, for the population as a whole these symptoms must not be regarded important contributors to the hangover state, and it is unlikely that important sex differences will be observed among these symptoms.

Fourth, there are several factors that contribute to hangover severity that were not included in the current analysis. These are discussed in various review articles on alcohol hangover [93,94,95], although no possible relationship with sex differences was suggested. Examples of factors that may impact the presence and severity of hangover symptoms are sleep duration and quality, physical activity while drinking, psychological state, congener content and type of drink, and having a family history of alcoholism. In the current analysis, these factors were not taken into account. Future research should examine the impact of factors that may aggravate hangover severity on possible sex differences. It may, for example, be possible that men may consume more congener rich drinks than women, or vice versa, which may explain observed gender differences. Alternatively, women may be more engaged in dancing during a drinking session than men or may consume more non-alcoholic drinks during a night out. Currently, this information is lacking from the scientific literature.

Finally, future research should investigate possible sex differences in memory, cognitive, and psychomotor functioning. The latter is important as the relative absence of important sex differences in the presence and severity of hangover symptoms does not imply that no sex differences may exist in performance outcomes and the conductance of daily life activities.

## 5. Conclusions

In conclusion, at the same BAC level, young adult male and female social drinkers experience hangover symptoms in comparable ways. No relevant sex differences were observed in the presence and severity of hangover symptoms across all eBAC levels. Future research should extend this investigation to other age groups.

## Figures and Tables

**Table 1:** Demographics.

**Demographics**	**Overall**	**Men**	**Women**	***p*-Value**
	(*n* = 1765)	(*n* = 895)	(*n* = 870)	
Age (years)	20.9 (2.3)	21 (2.4)	20.7 (2.2)	0.003 *
Body weight (kg)	71.9 (12.2)	77.4 (11.3)	66.3 (10.5)	0.000 *
Alcoholic drinks per week	13.2 (9.8)	16.6 (10.6)	9.7 (7.5)	0.000 *
**Latest Past Month Drinking Session that Produced a Hangover**				
Number of alcoholic drinks	12.1 (4.7)	14.1 (5)	10.0 (3.4)	0.000 *
Duration of the drinking session (h)	5.8 (2.0)	6.0 (2)	5.5 (1.9)	0.000 *
eBAC (%)	0.18 (0.08)	0.18 (0.08)	0.18 (0.08)	0.321
Overall hangover severity	6.1 (1.9)	6.1 (1.8)	6.2 (1.9)	0.367

Mean (SD) are shown. Differences are considered significant if *p* < 0.05 (indicated with *). Abbreviation: eBAC = estimated blood alcohol concentration.

**Table 2:** Presence and severity of individual hangover symptoms after alcohol consumption and reaching an eBAC 0% ≤ eBAC < 0.4%.

Symptoms	Mean (SD) All	% All	Mean (SD) Men	% Men	Mean (SD) Women	% Women
Headache	5.7 (2.5)	91%	5.7 (2.4)	90.7%	5.8 (2.5)	91.3%
Nausea	5.6 (2.6)	86%	5.2 (2.5)	84.1%	5.9 (2.7) *	87.5%
Concentration problems	6.0 (2.4)	91.1%	6.1 (2.4)	92.2%	5.8 (2.5)	90.1%
Regret	4.5 (2.7)	58.9%	4.4 (2.7)	57.1%	4.6 (2.7)	60.2%
Sleepiness	6.6 (2.3)	94.9%	6.4 (2.3)	94.3%	6.8 (2.2) *	95.3%
Heart pounding	4 (2.4)	35.5%	4 (2.4)	35.7%	4.0 (2.5)	35.3%
Vomiting	5.3 (3.2)	26%	4.8 (3.1)	23.5%	5.6 (3.2) *	28.1%
Tired	6.9 (2.2)	97.7%	6.6 (2.2)	96.8%	7.1 (2.2) *	98.4%
Shivering	4.2 (2.5)	45.7%	4.2 (2.5)	41.4%	4.3 (2.5)	49.1%*
Clumsy	4.9 (2.4)	72.2%	4.8 (2.4)	72.4%	4.9 (2.4)	72.1%
Weakness	5.4 (2.4)	85.2%	5.1 (2.4)	83.4%	5.6 (2.4) *	86.7%
Dizziness	4.4 (2.6)	62.3%	4.1 (2.4)	61%	4.6 (2.7) *	63.3%
Apathy	5.8 (2.5)	80.1%	5.6 (2.5)	78.3%	5.9 (2.6)	81.5%
Sweating	4.3 (2.4)	54.8%	4.3 (2.4)	58.5%	4.3 (2.5)	51.7%*
Stomach Pain	4.4 (2.5)	57.3%	4.3 (2.4)	54.4%	4.5 (2.5)	59.7%
Confusion	4.2 (2.4)	46.3%	4.1 (2.4)	49.9%	4.2 (4.5)	43.4%*
Light sensitivity	4.1 (2.4)	52.2%	3.9 (2.3)	52.4%	4.3 (2.4) *	52%
Thirst	6.7 (2.4)	94.5%	6.7 (2.4)	95%	6.6 (2.4)	94.1%
Heart racing	4.2 (2.5)	41.6%	4.2 (2.5)	45.7%	4.1 (2.5)	38.3%*
Anxiety	3.2 (2.4)	18.3%	3.2 (2.4)	17.4%	3.2 (2.4)	19.1%
Depression	3.8 (2.5)	29.9%	3.9 (2.5)	28.9%	3.8 (2.5)	30.6%
Reduced appetite	5.5 (2.6)	70.1%	5.5 (2.6)	69.9%	5.5 (2.6)	70.3%

Significant differences (*p* < 0.002, after Bonferroni correction) between men and women are indicated with *.

**Table 3:** Presence and severity of individual hangover symptoms after alcohol consumption and reaching an eBAC 0% ≤ eBAC < 0.08%.

Symptoms	Mean (SD) All	% All	Mean (SD) Men	% Men	Mean (SD) Women	% Women
Headache	5.5 (2.3)	90.3%	5.7 (2.2)	89%	5.4 (2.5)	91.4%
Nausea	4.9 (2.6)	82.2%	4.9 (2.6)	80%	5 (2.6)	84.1%
Concentration problems	5.3 (2.4)	85.4%	5.4 (2.2)	86%	5.3 (2.6)	84.9%
Regret	4.1 (2.8)	50.0%	4.2 (2.8)	52.9%	4.1 (2.8)	47.4%
Sleepiness	6.2 (2.3)	92.7%	5.8 (2.3)	91.1%	6.5 (2.3)	94.1%
Heart pounding	3.8 (2.5)	30.2%	3.8 (2.7)	37.5%	3.8 (2.2)	23.7%
Vomiting	4.5 (3.1)	20.5%	4.4 (3.2)	22.1%	4.7 (3.1)	19.1%
Tired	6.3 (2.4)	97.2%	5.9 (2.3)	95.6%	6.6 (2.4)	98.7%
Shivering	3.8 (2.2)	34.4%	3.9 (2.4)	36%	3.6 (2.0)	32.9%
Clumsy	4.3 (2.3)	56.6%	4.3 (2)	56.6%	4.3 (2.5)	56.6%
Weakness	4.8 (2.4)	74.3%	4.7(2.4)	72.8%	5 (2.4)	75.7%
Dizziness	3.9 (2.4)	52.8%	3.8 (2.3)	55.9%	4 (2.6)	50.0%
Apathy	5.5 (2.6)	74.2%	5.3 (2.6)	71.9%	5.8 (2.5)	76.3%
Sweating	4.1 (2.4)	44.6%	4.4 (2.4)	48.5%	3.8 (2.5)	41.1%
Stomach Pain	3.8 (2.2)	55.4%	3.8 (2.4)	53.7%	3.8 (2.1)	57.0%
Confusion	3.6 (2.5)	36.8%	3.8 (2.4)	41.2%	3.4 (2.6)	32.9%
Light sensitivity	4 (2.3)	42%	3.7 (2.3)	44.1%	4.3 (2.2)	40.1%
Thirst	6 (2.5)	90.9%	5.9 (2.4)	88.9%	6.1 (2.5)	92.7%
Heart racing	4 (2.6)	30.3%	4.2 (2.8)	36.3%	3.8 (2.4)	25%
Anxiety	3.1 (2.4)	12.9%	3.5 (2.8)	14.7%	2.6 (1.8)	11.3%
Depression	3.7 (2.3)	24.6%	3.9 (2.4)	25.9%	3.4 (2.1)	23.3%
Reduced appetite	4.6 (2.6)	60.4%	4.3 (2.5)	56.6%	4.8 (2.6)	63.8%

No significant differences (*p* < 0.002, after Bonferroni correction) were found between men and women.

**Table 4:** Presence and severity of individual hangover symptoms after alcohol consumption and reaching an eBAC of 0.08% ≤ eBAC < 0.11%.

Symptoms	Mean (SD) All	% All	Mean (SD) Men	% Men	Mean (SD) Women	% Women
Headache	5.6 (2.4)	88.8%	5.7 (2.3)	89.3%	5.4 (2.5)	88.4%
Nausea	5.3 (2.6)	83%	4.7 (2.5)	83.5%	5.8 (2.6) *	82.6%
Concentration problems	5.4 (2.4)	88.4%	5.9 (2.2)	87.6%	5.1 (2.5)	89%
Regret	4.4 (2.6)	59.8%	4.5 (2.7)	57%	4.3 (2.6)	61.9%
Sleepiness	6.4 (2.4)	92.8%	6.1 (2.4)	91.7%	6.5 (2.4)	93.5%
Heart pounding	3.5 (2.3)	32.6%	3.6 (2.4)	29.8%	3.4 (2.2)	34.8%
Vomiting	5.2 (3.1)	21.4%	4 (3.1)	15.7%	5.8 (3.0)	25.8%
Tired	6.7 (2.3)	96.4%	6.6 (2.2)	95%	6.8 (2.4)	97.4%
Shivering	4.2 (2.7)	42%	3.5 (2.5)	35.5%	4.6 (2.7)	47.1%
Clumsy	4.5 (2.3)	69.1%	4.6 (2.4)	68.6%	4.3 (2.3)	69.5%
Weakness	5.2 (2.3)	86.2%	4.9 (2.3)	84.3%	5.3 (2.3)	87.7%
Dizziness	4.3 (2.6)	58%	4.3 (2.3)	55.4%	4.2 (2.7)	60%
Apathy	5.5 (2.5)	77.5%	5.5 (2.3)	71.1%	5.5 (2.6)	82.5%
Sweating	3.9 (2.3)	48.6%	3.6 (2.2)	49.6%	4.2 (2.4)	47.7%
Stomach Pain	4.4 (2.6)	52.6%	4.1 (2.3)	53.3%	4.7 (2.8)	51.9%
Confusion	3.8 (2.2)	37.1%	3.7 (2.1)	40.5%	3.9 (2.4)	34.4%
Light sensitivity	4.1 (2.4)	50%	3.9 (2.3)	50.4%	4.2 (2.6)	49.7%
Thirst	6.4 (2.5)	93.1%	6.5 (2.5)	94.2%	6.3 (2.5)	92.3%
Heart racing	3.4 (2.2)	38.9%	3.4 (2.1)	43%	3.4 (2.3)	35.7%
Anxiety	2.9 (2.3)	16.4%	2.7 (1.9)	15.8%	3 (2.7)	16.9%
Depression	3.7 (2.5)	30.4%	3.4 (2.4)	25.6%	3.9 (2.5)	34.2%
Reduced appetite	5.6 (2.5)	62%	5.4 (2.6)	61.2%	5.8 (2.5)	62.6%

Significant differences (*p* < 0.002, after Bonferroni correction) between men and women are indicated with *.

**Table 5:** Presence and severity of individual hangover symptoms after alcohol consumption and reaching an eBAC of 0.11% ≤ eBAC < 0.2%.

Symptoms	Mean (SD) All	% All	Mean (SD) Men	% Men	Mean (SD) Women	% Women
Headache	5.8 (2.4)	91.1%	5.6 (2.5)	90.5%	5.9 (2.4)	91.7%
Nausea	5.7 (2.7)	86.4%	5.3 (2.7)	84.4%	6 (2.6) *	88.1%
Concentration problems	6.0 (2.4)	92.3%	6.1 (2.3)	94.6%	6 (2.4)	90.5%
Regret	4.5 (2.7)	61.1%	4.2 (2.6)	58.8%	4.7 (2.7)	62.9%
Sleepiness	6.6 (2.3)	95.7%	6.4 (2.3)	96.3%	6.8 (2.2)	95.2%
Heart pounding	3.9 (2.4)	36.3%	4 (2.4)	36.2%	3.9 (2.4)	36.3%
Vomiting	5.4 (3.1)	26%	5 (3.1)	23.5%	5.6 (3.1)	28.1%
Tired	7 (2.2)	97.3%	6.8 (2.2)	96.3%	7.2 (2.1) *	98.1%
Shivering	4.2 (2.6)	47.6%	4.3 (2.5)	42%	4.2 (2.7)	51.9%*
Clumsy	4.9 (2.4)	72.2%	5 (2.4)	73.7%	4.9 (2.5)	71.1%
Weakness	5.4 (2.4)	86.3%	5.1 (2.4)	83.7%	5.7 (2.4) *	88.4%
Dizziness	4.4 (2.5)	62.9%	4.0 (2.4)	62.4%	4.8 (2.6) *	63.4%
Apathy	5.8 (2.5)	81.3%	5.6 (2.5)	79%	6 (2.5)	83.1%
Sweating	4.2 (2.4)	55.4%	4.3 (2.4)	59.7%	4.2 (2.4)	52.1%
Stomach Pain	4.5 (2.5)	57.6%	4.3 (2.5)	54.8%	4.6 (2.5)	59.8%
Confusion	4 (2.4)	47.5%	3.9 (2.4)	53%	4.2 (2.4)	43.2%*
Light sensitivity	4.1 (2.5)	53.6%	3.8 (2.4)	55%	4.4 (2.5)	52.6%
Thirst	6.7 (2.3)	95.3%	6.8 (2.3)	95.9%	6.7 (2.3)	94.9%
Heart racing	4.2 (2.5)	41.2%	4.3 (2.5)	45.4%	4.2 (2.5)	37.9%
Anxiety	3.4 (2.4)	20%	3.3 (2.4)	19%	3.4 (2.4)	20.9%
Depression	3.8 (2.5)	31.5%	3.9 (2.5)	31.6%	3.8 (2.5)	31.5%
Reduced appetite	5.5 (2.6)	72.5%	5.5 (2.6)	73.1%	5.6 (2.6)	72.1%

Significant differences (*p* < 0.002, after Bonferroni correction) between men and women are indicated with *.

**Table 6:** Presence and severity of individual hangover symptoms after alcohol consumption and reaching an eBAC of 0.2% ≤ eBAC < 0.3%.

Symptoms	Mean (SD) All	% All	Mean (SD) Men	% Men	Mean (SD) Women	% Women
Headache	5.7 (2.6)	92.2%	5.8 (2.5)	92.8%	5.6 (2.6)	91.7%
Nausea	5.6 (2.6)	88.8%	5.2 (2.4)	86%	6 (2.7) *	91.1%
Concentration problems	6.1 (2.4)	93.1%	6.3 (2.4)	93.8%	6 (2.4)	92.5%
Regret	4.6 (2.7)	60.8%	4.5 (2.6)	59.9%	4.7 (2.8)	61.5%
Sleepiness	6.8 (2.1)	95.1%	6.7 (2.1)	93.4%	6.9 (2.1)	96.4%
Heart pounding	4.3 (2.5)	37.4%	4.4 (2.4)	35.4%	4.3 (2.5)	39%
Vomiting	5.5 (3.4)	27%	4.8 (3.1)	24%	6 (3.5)	29.5%
Tired	7 (2.1)	98.6%	6.7 (2.1)	98.3%	7.2 (2.1) *	98.9%
Shivering	4.4 (2.4)	48.6%	4.3 (2.5)	44.2%	4.4 (2.3)	52.2%
Clumsy	5.1 (2.4)	79%	4.9 (2.5)	78.9%	5.2 (2.4)	79.1%
Weakness	5.5 (2.4)	87.7%	5.3 (2.3)	86.9%	5.7 (2.4)	88.3%
Dizziness	4.4 (2.7)	66.7%	4.2 (2.5)	62.7%	4.6 (2.8)	69.9%
Apathy	5.7 (2.5)	81.9%	5.7 (2.4)	83.2%	5.8 (2.6)	80.8%
Sweating	4.5 (2.5)	58.4%	4.5 (2.4)	62.3%	4.5 (2.6)	55.2%
Stomach Pain	4.5 (2.5)	59.5%	4.4 (2.4)	54.6%	4.5 (2.6)	63.5%
Confusion	4.6 (2.5)	51.4%	4.5 (2.5)	52.6%	4.6 (2.6)	50.4%
Light sensitivity	4.2 (2.3)	55.2%	4.2 (2.2)	53.4%	4.3 (2.4)	56.7%
Thirst	6.8 (2.4)	95.5%	6.9 (2.3)	96.9%	6.8 (2.4)	94.4%
Heart racing	4.5 (2.5)	47.0%	4.6 (2.5)	49.5%	4.4 (2.6)	45%
Anxiety	3 (2.3)	19.3%	2.9 (2.3)	17.8%	3.1 (2.2)	20.6%
Depression	3.9 (2.4)	29.6%	4 (2.4)	29.8%	3.8 (2.5)	29.5%
Reduced appetite	5.5 (2.5)	73.3%	5.7 (2.4)	73.6%	5.4 (2.6)	73.1%

Significant differences (*p* < 0.002, after Bonferroni correction) between men and women are indicated with *.

**Table 7:** Presence and severity of individual hangover symptoms after alcohol consumption and reaching an eBAC of 0.3% ≤ eBAC < 0.4%.

Symptoms	Mean (SD) All	% All	Mean (SD) Men	% Men	Mean (SD) Women	% Women
Headache	5.9 (2.5)	90.6%	5.5 (2.5)	89.2%	6.3 (2.4)	91.8%
Nausea	6 (2.6)	84.3%	5.6 (2.5)	84%	6.4 (2.6)	84.5%
Concentration problems	6.4 (2.4)	89.8%	6.6 (2.4)	90%	6.3 (2.5)	89.7%
Regret	5.2 (2.8)	51.7%	5.4 (2.8)	45.1%	5.1 (2.8)	57.3%
Sleepiness	6.8 (2.4)	96.1%	6.4 (2.5)	95.2%	7 (2.3)	96.9%
Heart pounding	4.5 (2.6)	37.4%	4.1 (2.5)	40.2%	5 (2.6)	35.1%
Vomiting	5.3 (3.3)	38.5%	5.2 (3.2)	35.4%	5.5 (3.3)	41.2%
Tired	7 (2.4)	98.9%	6.7 (2.4)	98.8%	7.3 (2.3)	99%
Shivering	4.4 (2.7)	47.8%	4.3 (2.9)	46.3%	4.4 (2.6)	49%
Clumsy	5 (2.6)	77.7%	4.7 (2.7)	73.2%	5.2 (2.5)	81.4%
Weakness	5.8 (2.4)	85.4%	5.4 (2.3)	85.2%	6.2 (2.4)	85.6%
Dizziness	4.6 (2.5)	64.2%	4.3 (2.4)	64.6%	4.9 (2.6)	63.9%
Apathy	6.3 (2.5)	79.9%	6.3 (2.5)	78%	6.3 (2.5)	81.4%
Sweating	4.6 (2.4)	63.7%	4.7 (2.5)	68.3%	4.6 (2.3)	59.8%
Stomach Pain	4.7 (2.5)	58.2%	4.7 (2.5)	53.8%	4.7 (2.5)	61.9%
Confusion	4.6 (2.4)	50.3%	4.7 (2.3)	51.2%	4.6 (2.5)	49.5%
Light sensitivity	4.2 (2.3)	52.2%	3.6 (2.1)	50.6%	4.6 (2.3)	53.6%
Thirst	7 (2.3)	93.9%	7 (2.2)	94%	7 (2.4)	93.8%
Heart racing	4.4 (2.5)	46.9%	4 (2.4)	53.7%	4.9 (2.6)	41.2%
Anxiety	3.6 (2.5)	16.3%	3.3 (2.6)	13.6%	3.8 (2.5)	18.6%
Depression	4.1 (2.7)	28.5%	4.3 (3)	20.7%	4.1 (2.6)	35.1%
Reduced appetite	6.2 (2.5)	72.6%	6.2 (2.4)	73.2%	6.1 (2.5)	72.2%

No significant differences (*p* < 0.002, after Bonferroni correction) were found between men and women.
