# Correction: Van Lawick van Pabst, A.E. et al. Sex Differences in the Presence and Severity of Alcohol Hangover Symptoms. *Journal of Clinical Medicine* 2019, *8*, 867

**DOI:** 10.3390/jcm8091308

**Published:** 2019-08-26

**Authors:** Albertine E. van Lawick van Pabst, Lydia E. Devenney, Joris C. Verster

**Affiliations:** 1 Division of Pharmacology, Utrecht University, 3584 CG Utrecht, The Netherlands; 2 School of Psychology, Life and Health Sciences Ulster University, Londonderry BT52 1SA, Northern Ireland, UK; 3 Centre for Human Psychopharmacology, Swinburne University, Melbourne VIC 3122, Australia

The paper [1] contains combined data from two studies [2,3] and none of the *n* = 2446 subjects were excluded from the statistical analysis. During the data analysis, the authors accidentally selected data from one study only [2], and used these to calculate the number of men and women for each estimated blood alcohol concentration (eBAC) range. The statement in the paper that “…those who reported no past month hangover were omitted from the analysis (*n* = 681, 27.6%)” is therefore incorrect. The omitted subjects were in fact the participants from the other study [3], in the combined dataset. The reported number of men and women, both overall and for each eBAC group, therefore needs to be corrected.

The authors would like to make the following corrections to the published paper [1]:

(1) In the result section replace:

“From the *n* = 2446 subjects that completed the survey, those who reported no past month hangover were omitted from the analysis (*n* = 681, 27.6%). Thus, data on the presence and severity of hangover symptoms from *n* = 1765 subjects (*n* = 895 men and *n* = 870 women) were compared.

“All *n* = 2446 subjects completed the survey (*n* = 1094 men and *n* = 1352 women).”.

(2) The overall number of men and women in the abstract and text, “53.7% men and 47.3% women” should be replaced by “55.3 men and 44.7% women”.

(3) Correct the numbers of men and women, overall, and for each eBAC range, as follows:

Overall: replace: “1765 subjects (*n* = 895 men and *n* = 870 women)” by “2446 subjects (*n* = 1094 men and *n* = 1352 women)” in the text and in Table 1.

0% ≤ eBAC < 0.08%: replace: “*n* = 91 men and *n* = 68 women” by “*n* = 136 men and *n* = 152 women” in the text.

0.08% ≤ eBAC < 0.11%: replace: “*n* = 95 men and *n* = 93 women” by “*n* = 121 men and *n* = 155 women” in the text.

0.11% ≤ eBAC < 0.20%: replace: “*n* = 403 men and *n* = 383 women” by “*n* = 462 men and *n* = 588 women” in the text.

0.20% ≤ eBAC < 0.30%: replace: “*n* = 244 men and *n* = 264 women” by “*n* = 292 men and *n* = 360 women” in the text.

0.30% ≤ eBAC < 0.40%: replace: “*n* = 62 men and *n* = 62 women” by “*n* = 83 men and *n* = 97 women” in the text.

(4) The reported percentage of subjects with an eBAC below 0.11%, “18.7% of all women and 20.6% of all men” should be replaced by “22.7% of all women and 23.5% of all men”.

The authors would like to apologize for any inconvenience caused to the readers by these changes. The changes do not affect the scientific results presented in the paper or its interpretation. The manuscript will be updated and the original will remain online on the article webpage.
